# Effects of Curcumin on Growth Performance, Ruminal Fermentation, Rumen Microbial Protein Synthesis, and Serum Antioxidant Capacity in Housed Growing Lambs

**DOI:** 10.3390/ani13091439

**Published:** 2023-04-23

**Authors:** Guangyuan Tian, Xuanzi Zhang, Xiaoyan Hao, Jianxin Zhang

**Affiliations:** College of Animal Science, Shanxi Agriculture University, Jinzhong 030801, China

**Keywords:** curcumin, growth performance, ruminal fermentation, microbial protein, antioxidant, lambs

## Abstract

**Simple Summary:**

The strong antioxidant properties of curcumin (CUR) could alleviate oxidative stress suffered by housed growing lambs, thus promoting growth. This study shows that dietary CUR can promote rumen fermentation, enhance rumen microbial activity, and promote microbial protein synthesis. However, high doses of CUR could inhibit the number of *Fibrobacter succinogenes*, thus affecting the weight gain of lambs. Our research results provide evidence for the addition of CUR to certain ruminant diets.

**Abstract:**

This experiment was conducted to investigate growth performance, ruminal fermentation, rumen microbial protein synthesis, and serum antioxidant capacity with different doses of curcumin (CUR) included in the diet of housed growing lambs. Forty-eight four-month-old Dorper × Thin-tailed Han F_1_ crossbred male lambs (body weight = 20.89 ± 1.15 kg, age = 120 ± 10 days; mean ± SD) were randomly divided into four groups for a single-factor, completely randomized experiment. Treatments comprised the following: the basal diet supplemented with 0 (Control), 300 mg/kg (300 CUR), 600 mg/kg (600 CUR), or 900 mg/kg (900 CUR) CUR, respectively. The results showed that dietary CUR increased average daily gain (ADG), and the 300 CUR group evidenced the highest value. There were no significant effects on dry matter intake (DMI) and DMI/ADG. Lambs in the 300 CUR group showed higher totals of volatile fatty acids (VFA) and acetate than other groups, while decreased valerate was observed with supplementary CUR. The ruminal pH and ammonia N (NH_3_-N) concentration decreased with increasing CUR, with the greatest effect in the 300 CUR group. The quadratic effects were found in pectinase, carboxymethyl cellulose, and protease, with the greatest value in the 300 CUR group. The microbial populations of total bacteria and *Ruminococcus albus* also responded quadratically, and the methanogens, protozoan, and *Fibrobacter succinogenes* populations decreased linearly with increasing CUR. Lambs receiving additional CUR showed increased *Prevotella ruminicola* population. Microbial protein (MCP) synthesis was promoted by supplementary CUR. As supplementation with CUR increased, the serum activity of total antioxidant capacity (T-AOC), superoxide dismutase (SOD), and glutathione peroxidase (GSH-Px) was enhanced, with the greatest value in the 300 CUR group. In conclusion, dietary CUR improved ruminal fermentation, promoted rumen microbial protein (MCP) synthesis, and enhanced serum antioxidant activity, as well as promoting growth performance in housed growing lambs.

## 1. Introduction

Antibiotics are routinely used to treat diseases in livestock production. However, studies show antibiotic resistance, drug residues, and other problems not only limit the development of livestock production but also threaten human health [1]. As a result, there is an increasing global focus on natural, green, and multifunctional feed additive products to alternative antibiotics such as plant extracts, vitamin E, vitamin C, synthetic phenolic compounds, and so on [2]. China is the country with the largest number of natural plant species in the world and also the earliest country to use plant extracts as feed additives. Additionally, plant extracts offer the advantage of fewer side effects than other feed additive products [3].

Curcumin (CUR) is a polyphenolic natural pigment extracted from plants in the ginger family. CUR is composed of the phenolic hydroxyl groups, heptadiene chain, and diketone moiety [4]. Since multiple reactive groups in the structure of CUR, such as the phenol hydroxyl group, carbonyl group, double bond, and so on, it has antibacterial [5], anti-inflammatory [6], antioxidant [7], and other functions. Numerous studies show plant polyphenols could affect the volatile fatty acids (VFA) of ruminants [8], improve the utilization of nitrogen [9], regulate the hydrogenation of fatty acids [10], and affect the activity and quantity of rumen microorganisms [11]. Rumen microbial proteins (MCP) are synthesized by rumen microorganisms from rumen degradable proteins (RDP). Therefore, dietary supplementation of plant polyphenols could promote microbial protein synthesis in ruminants. Marcon et al. [12] added 100, 200, and 300 mg/kg CUR to lambs’ basal diets and demonstrated increased ADG and carcass yield with the dose of 300 mg/kg. Moreover, they also found that the greatest dosage of CUR for weight gain was 315.1 mg/kg via nonlinear regression analysis. Jiang et al. [13] showed that dietary supplementation of 450 and 900 mg/kg CUR could also improve the antioxidant capacity and immune response of lambs. Based on previous extensive animal and human studies, high doses of CUR had no toxic effects. Therefore, this experiment was conducted to increase the CUR addition in order to reveal the optimal dose in lambs.

The present study aimed to investigate the effects of dietary curcumin on growth performance, ruminal fermentation, rumen microbial protein synthesis, and serum antioxidant capacity in housed growing lambs.

## 2. Materials and Methods

### 2.1. Animals, Experimental Design, and Management

This study was approved by the Animal Care and Ethical Committee of Shanxi Agricultural University (Approval No.: SXAU-EAW-2019C012004) and conducted at a commercial farm of Youyu Xianghelingshang from August to November 2021. A total of 48 Dorper × Thin-tailed Han crossbred male lambs averaging 120 ± 10 days of age and 20.89 ± 1.15 kg in body weight were used in the study. The animals were randomly allocated into four groups (n = 12) and fed the basal diet supplemented with 0 (Control), 300 (300 CUR), 600 (600 CUR), and 900 mg/kg (900 CUR) CUR, respectively. The CUR (purity ≥ 98%) was purchased from Wuxi Shiji Biotechnology Co., Ltd. (Wuxi, China). The daily addition of CUR was adjusted based on the weight of the basal diet consumed the previous day and was mixed with 80 g of powdery base diet then top-dressed in the morning feeding. The basal diet comprised a 60:40 concentrate: the forage ratio was formulated based on NRC (2007) and a total mixed pelleted feed. The chemical composition and ingredients of the experimental diets are shown in Table 1. 

The experiment consisted of three periods and lasted 90 days: d 1 through d 15 served as an adaptation phase; d 16 through d 60 served as a feeding trial phase; and d 61 through d 90 served as a digestion and metabolism trial phase. During the adaptation and feeding trial phase, the animals were housed individually in stalls of 3 × 0.8 m and fed a pelleted TMR twice daily at 0730 h and 1730 h. The animals had free access to clean drinking water. 

### 2.2. Data Collection and Sampling of Blood and Rumen Fluid

Initial and final body weights were taken at the start (day 1) and end (day 60) of the feeding trial phase before morning feeding. During the feeding trial phase, feed intakes and refusals were recorded daily for the calculation of average daily feed intake (ADFI), average daily gain (ADG), and ratio of feed to gain (F/G). At the end of the feeding trial phase, blood samples were collected from the jugular vein before morning feeding. Serum was obtained after centrifugation (15 min at 2000× *g*, 4 °C) and stored at −80 °C until analysis. On day 76, the ruminal fluid of each lamb was collected 3 h after supplying feed in the morning using an oral stomach tube. To avoid saliva contamination, the initially collected 100 mL of ruminal fluid was abandoned, and the subsequent 80 mL was retained.

### 2.3. Determination of Rumen Fermentation Parameters and Rumen Enzyme Activity

The ruminal fluid samples pH was determined by using an electric pH meter (PHS-3S). Immediately following, the samples were filtered through four layers of gauze and separated to centrifuge tubes, which were stored at −80 °C until analysis. The content of rumen volatile fatty acid (VFA) was determined using gas chromatography (Agilent 7890B; Agilent Technologies Ltd., Palo Alto, USA) based on the method described by Wang et al. [14]. The content of rumen ammonia N (NH_3_-N) was determined via an ultraviolet spectrophotometer (UV1100; Techcomp Instrument Co., Ltd., Shanghai, China) based on the method described by (Bradford et al., 1976). The activity of cellobiase, pectinase, Carboxymethyl cellulose, Xylanase, and α-amylase of the ruminal fluid was determined based on the method described by Agarwal et al. [15].

### 2.4. Determination of Purine Derivatives and Microbial Proteins

During the sampling collection period of the digestion and metabolism trial, the urine was collected by a funnel into 100 mL of 10% sulfuric acid (H_2_SO_4_) and was stored at −20 °C until analysis. The content of allantoin in urine was determined by using an ultraviolet spectrophotometer (UV1100; Techcomp Instrument Co., Ltd., Shanghai, China) according to the method described by Chen et al. [16]. The contents of uric acid were determined using Uric acid kits (Nanjing Jiancheng bioengineering institute Co., Ltd., Nanjing, China). The urinary purine derivatives (PD) and microbial protein (MCP) were estimated according to the method described by Chen et al. [16].

### 2.5. Microbial DNA Extraction and RT-qPCR

Total microbial DNA were extracted from ruminal fluid using the repeated bead-beating method described by Yu et al. [17]. The extracted air-dried DNA was added to 50 μL TE buffer to dissolve DNA adequately. Subsequently, the concentration and quality of DNA were measured via a Nano Drop 2000 spectrophotometer (Thermo Scientific, Nano Drop Technologies, Rockland, DE, USA). The target microbial l primers were provided by Beijing Huada Gene Technology Co., Ltd. (Beijing, China), and the set sequences are listed in Table 2. The standard curves for target micro-organisms were established by ten-fold serial dilution [18]. Real-time PCR was detected for target genes using SYBR^®^ Primic Ex TaqTM II (TliRNaseH Plus) kits (TaKaRa, Dalian, China). The real-time PCR reaction system is as follows: 2 μL of DNA template; 10.0 μL SYBR^®^ Primic Ex TaqTM (TliRNaseH Plus); 0.4 μL PCR Forward Primer (10 μmol/L); 0.4 μL PCR Reverse Primer (10 μmol/L); and 7.2 μL Nuclease-Free Water. The PCR conditions were as follows: one cycle of 50 °C for 2 min and 95 °C for 2 min for initial denaturation; and 40 cycles of 95 °C for 15 s and 60 °C for 1 min for primer annealing and product extension using the method of Wu et al. [19].

### 2.6. Determination of Serum Antioxidant Capacity and Oxidative Stress

The serum activity of total antioxidant capacity (T-AOC), superoxide dismutase (SOD), glutathione peroxidase (GSH-Px), catalase (CAT), and malonaldehyde (MDA) were determined using the Corresponding kits (Shanghai Meilian Biotechnology Co., Ltd., Shanghai, China).

### 2.7. Statistical Analysis

All experimental data were initially collated using Excel version 2007, and one-way ANOVA was performed by SPSS (version 22.2, IBM SPSS Statistics, NY, USA). When significant differences were observed, multiple comparisons were performed using Duncan’s test and linear and quadratic regression analysis. Dietary concentration of CUR represented the treatment effect. Individual lambs were regarded as experimental units. Linear and quadratic regression analysis were used to describe the treatment effect of CUR dose so as to more accurately determine the optimal dosage under the conditions of this study. In addition, total weight gain during 1 to 60 days through the nonlinear regression method came up with the optimum level of CUR. The data were expressed as means and pooled SEM. A significant level of *p* ≤ 0.05 was used.

## 3. Results

### 3.1. Growth Performance

As shown in Table 3, there were no significant differences in initial body weight, DMI, and DMI/ADG between control and experimental groups. Quadratic (*p* < 0.050) effects were observed on final body weight and ADG with relatively higher values in the 300 CUR group.

**Figure 1:**
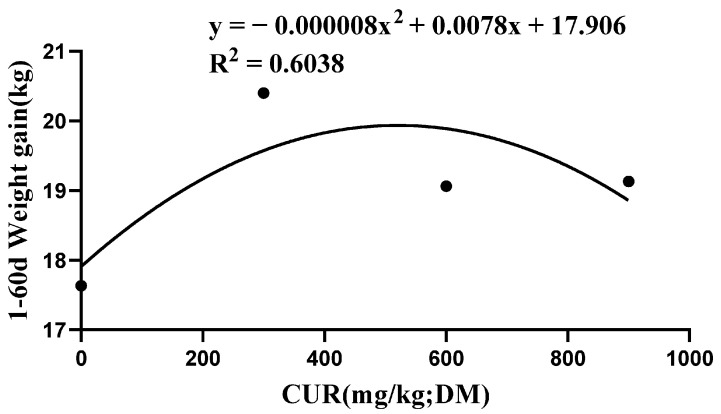
Effects of CUR in total weight gain during 1 to 60 days in housed growing lambs.

As shown in Figure 1, the weight gain curve was obtained through nonlinear regression analysis. In the figure, the abscissa is the level of curcumin supplementation, and the ordinate is the total weight gain of lambs during one to sixty days. The inflection point of the curve is x = 487.5, and the optimal supplemental dose can be inferred to be 487.5 mg/kg.

### 3.2. Ruminal pH, VFA and NH_3_-N

The results of ruminal pH, the concentration of VFA, and NH_3_-N were presented in Table 4. No significant difference was observed for the concentration of propionate, butyrate, isovalerate and acetate/propionate. Quadratic responses were found in pH (*p* = 0.008), total VFA (*p* = 0.024), and acetate (*p* = 0.009) with the greater values in the 300 CUR group. A linearly (*p* = 0.023) decreased response was observed for valerate with increasing CUR. Dietary CUR also affected the concentration of NH-N_3_ with a quadratic (*p* = 0.003) response, and the 300 CUR group had the lowest value of the groups.

### 3.3. Ruminal Enzyme Activity and Microbial Population

The ruminal enzyme activity and microbial population are shown in Table 5. No significant difference was observed for the activities of cellobiase, xylanase, and α-amylase. The quadratic effects were found in pectinase (*p* < 0.001), carboxymethyl cellulose (*p* = 0.013), and protease (*p* < 0.001) with the greatest value in the 300 CUR group. The population of total bacteria (*p* < 0.001) and *Ruminococcus albus* (*p* = 0.001) also responded quadratically. The microbial population of methanogens (*p* = 0.011) and protozoan (*p* = 0.022) decreased linearly with increasing CUR addition. A linear (*p* < 0.001) decrease in *Fibrobacter succinogenes* occurred with the lowest value in the 900 CUR group since the increase of CUR addition. Both the linear (*p* = 0.023) and quadratic (*p* = 0.032) effects were observed significantly in *Prevotella ruminicola* as the levels of CUR were increased in the diet. However, the *Ruminococcus flavefaciens* and *Ruminobacter amylophilus* populations were not affected by increasing CUR inclusion.

### 3.4. Purine Derivatives and Microbial Protein Synthesis

The estimation of purine derivates (PD) and MCP are presented in Table 6. These quadratic (*p* ≤ 0.021) effects were found in allantoin, xanthine + hypoxanthine, total PD, exogenous purines absorption, and MCP with relatively higher values in the 300 CUR group.

### 3.5. Serum Antioxidant Activity

The serum antioxidant capacity is shown in Table 7. There were quadratic trends on T-AOC ((*p* = 0.001), SOD (*p* = 0.004), and GSH-Px (*p* = 0.005) with the greatest values in the 300 CUR group. However, the activity of CAT and MDA showed no significant difference by supplementation with CUR.

## 4. Discussion

Feng et al. [20] reported that CUR had the defect of low bioavailability due to intrinsic hydrophobicity, chemical instability, and photodegradation. Therefore, some special methods were used to improve its bioavailability, such as preparation by nanotechnology [21] in combination with piperine [22] and encapsulation in microcapsules [23]. However, compared with metabolic products of tetrahydrocurcumin and octahydrocurcumin, CUR shows better antioxidant capacity, anti-inflammatory properties, and more stable chemical structures than previous research indicated [24]. In addition, CUR could maintain the health of the body by regulating the composition of the gastrointestinal microbiota [25]. These results indicated that CUR has natural antioxidants that do not rely on bioavailability, but rather they are metabolized into more stable metabolites in the animal’s gastrointestinal system to play a positive role. 

This experiment showed that CUR addition increased ADG, a finding consistent with previous research [26]. It is possible that this is related to increased nutrient digestibility. Vorlaphim et al. [26] reported that dietary CUR in beef cattle decreased fecal nitrogen excretion and increased nitrogen absorption and retention, which indicated that supplementation with CUR could develop the digestibility of crude protein. Furthermore, dietary CUR promoted the digestibility of neutral detergent fiber to increase milk production based on previous research [27]. The content of acetate in rumen fluid was positively correlated with the digestibility of neutral detergent fiber [28]. These results indicated that with the increase of the content of acetate, the digestibility of neutral detergent fiber would be improved accordingly. CUR as a strong antioxidant also increased ADG. The polyphenols of CUR could combine with reactive oxygen species to clear free radicals in housed growing models. The serum antioxidant activity in this study was promoted with CUR addition, which was in agreement with the result of growth performance. Additionally, Xun et al. [29] found that dietary CUR in piglets increased the ratio of ileal villus height to crypt depth, improved ileal mucosal morphology, and repaired the intestinal damage caused by *E. coli*. That finding indicated that the addition of CUR can promote the digestion and absorption of nutrients through a protective effect on the intestinal tract. Results from this study show that appropriate doses of CUR contributed to the growth and health of lambs. Compared with the 300 CUR group, however, the 600 and 900 groups showed less improvement in ADG, which might be associated with the decreased *Fibrobacter succinogenes* linearly in this study. As reported by Vorlaphim et al. [26], the digestibility of acid detergent fiber is reduced with increasing CUR addition in beef cattle. Thus, high doses of CUR might adversely affect the growth of lambs.

Ruminal pH was an important factor in response to the stability of the rumen internally [30]. Zhou et al. [31] reported that the appropriate ruminal pH values were from 6.56 to 6.95. In the current study, the ruminal pH value was 7.13 in the control group, which indicated that the growth and reproduction of rumen microflora were not favored under this condition. The decrease in ruminal pH values was in accordance with the increase of total VFA concentration by the addition of CUR, while carbohydrates in the diet were fermented in the rumen to produce VFA. Dietary CUR in humans activated bowel motility and promoted carbohydrate colonic fermentation, as reported by Akito et al. [32]. Moreover, Li et al. [33] reported that the increased nutrient digestibility contributed to the higher total VFA, which was the main source of energy for ruminants. Vitor et al. [34] reported that dietary CUR changed the activity of enzymes involved in adenosine triphosphate (ATP) metabolism, thus developing weight gain in lambs. The above study indicated that dietary CUR improved the energy metabolism of lambs and thus promoted growth. Li et al. [33] reported that the high levels of acetate related to the increased *Fibrolytic* micro-organism population and that the development of the amylolytic microorganisms contributed to the increased propionate. The *Ruminococcus albus*, *Ruminococcus flavefaciens*, *Fibrobacter succinogenes,* and *Butyrivibrio fibrisolvens* populations were the main fibrinolytic bacteria in the rumen with strong fibre degradation ability. In this study, dietary CUR increased the content of acetate, which was in agreement with the improvement of the total bacteria, *Ruminococcus albus* and *Fibrobacter succinogenes*. Likewise, that also was associated with the increased activity of pectinase and parboxymethyl cellulose. The protozoan decreased linearly with the addition of CUR, which was consistent with previous research [26]. This result also contributed to the lower values of VFA in the 600 and 900 CUR group compared with the 300 CUR group. Protozoan could produce approximately 10% of the VFA and digested 30% of fibre, as reported by Wu et al. [19]. Methanogens were symbiotic with protozoan in rumen [35], and one study showed that methane production decreased by 9% to 40% with the disappearance of the protozoan [36]. In the current study, the response of methanogens was in accordance with the change of the protozoan and ruminal pH, which indicated that dietary CUR in lambs could reduce methane production to protect the environment Several animal studies had shown that CUR had a positive effect on micro-organisms. The composition of the gut microbiota has been improved by CUR supplementation [37]. Another study concluded that supplementation CUR in a murine model of hyper-acute Th1-type ileitis following peroral infection with *Toxoplasma gondii* increased anti-inflammatory lactobacilli and bifidobacterial and decreased pro-inflammatory enterobacteria [38]. In this study, a linear decrease in *Fibrobacter succinogenes* occurred with the lower value in the 900 CUR group since the increase of CUR addition. Recent studies also had shown that certain plant secondary metabolites, such as the a dose of grape seed pro-anthocyanidins [39] and soluble substances of astragalus [40], could inhibit *Fibrobacter succinogenes* and *Butyrivibrio fibrisolvens.* However, *Butyrivibrio fibrisolvens* were unaltered with the CUR addition in this study. The difference between the results might be due to the dose of CUR not leading to significant inhibition.

The response of NH_3_-N concentration was consistent with the changes in MCP concentration, populations for protein degrading bacteria (*Prevotella ruminicola),* and the activity of protease, which was consistent with Vorlaphim et al. [26] research. Rumen micro-organisms degraded the mixture of dietary ammonia, free AA, and polypeptides to meet the NH_3_-N requirements for protein synthesis [41]. Increased rumen microflora and protease could provide more nitrogen sources for microbial protein synthesis. Moreover, the higher content of VFA could provide more energy. Firkins et al. [42] reported that rumen NH_3_-N came from feed protein degradation and the digestion of bacteria by protozoa. In this study, the decreased concentration for NH_3_-N also might be related to the reduction of protozoan with increasing the addition of CUR. Urinary PD came from duodenal absorption of nucleic acid purine, which was positively correlated with microbial protein synthesis [31]. The improvements of total PD and MCP could indicate that dietary CUR on lambs were more efficient in converting degradable dietary N to rumen microbial protein.

Under the housed growing model and although the productivity of livestock and poultry has been greatly improved, a series of problems, such as reproductive disorders, high morbidity rate, and decreased quality of animal products, have also emerged, which may be related to the level of oxidative stress in the animal organism [39]. Descalzo et al. [43] reported that fresh pasture contains high amounts of plant polyphenols. Therefore, compared to housed growing cattle, herding beef has a higher content of antioxidant substances. In the current study, a significant increase in antioxidant enzymes with supplemental CUR was observed, which was consistent with findings for pigs [44] and sheep [13]. The finding has also been reported that dietary CUR could enhance redox status and improve meat quality of intrauterine growth retardation for growing pigs via the Nrf2 signal pathway [45]. However, the antioxidant enzymes in the 600 and 900 CUR groups had lower values compared to the 300 CUR group. Previous studies had shown that curcumin could generate, via peroxidase action, oxidative phenoxy radicals, which could absorb large amounts of oxygen and produce ROS [46]. Thus, the antioxidant activity of CUR might exhibit dose dependence.

These results suggested that a higher level of CUR in rumen might interfere with microbial growth or metabolism, inhibit the fiber digestion, causing adverse effects on growth performance. 

## 5. Conclusions

Dietary CUR supplements of 300 mg/kg in lambs could improve growth performance, which is associated with the stimulatory impacts of CUR on rumen microbial growth and protein synthesis. CUR stimulated rumen fermentation to produce volatile fatty acids and provide energy for microbial protein synthesis. However, compared with the 300 group, the 600 and 900 groups showed less improvement on growth performance, a result which might be associated with the decreased *Fibrobacter succinogenes* linearly.

## Figures and Tables

**Table 1:** Composition and nutrient levels of the basal diet (air dry basis, %).

Items	Contents
Ingredients	
Soybean meal	7.00
Cottonseed meal	5.00
Corn germ meal	18.00
Sprayed corn husk	6.00
Corn	25.00
Peanut shell powder	10.00
Corn straw	14.00
Sunflower peel powder	10.00
Premix ^a^	5.00
Total	100.00
Nutrient levels	
Crude protein, CP	15.19
Ether extract, EE	2.20
Ash	9.30
Neutral detergent fiber, NDF	34.86
Acid detergent fiber, ADF	21.43
Metabolizable energy, ME; MJ/kg DM	9.53

^a^ The premix provided the following per kg of diets: Cu 15 Fe 55 mg, Zn 25 mg, Mn 40 mg, Se 0.3 mg, I 0.5 mg, Co 0.2 mg, VA 20 000 IU, VD 4 000 IU, VE 40 IU.

**Table 2:** Primer sequences for real-time PCR.

Target Species	Sequences, 5′–3′	Size, bp
Total bacteria	F:CGGTGAATACGTTCYCGG R:CGWTACCTTGTTACGACTT	123
Protozoan	F:GCTTTCGWTGGTAGTGTATT R:CTTGCCCTCYAATCGTWCT	223
Methanogens	F:TTCGGTGGATCDCARAGRGC R:GBARGTCGWAWCCGTAGAATCC	140
*Ruminococcus albus*	F:CCCTAAAAGCAGTCTTAGTTCG R:CCTCCTTGCGGTTAGAACA	176
*Ruminococcus flavefaciens*	F:ATTGTCCCAGTTCAGATTGC R:GGCGTCCTCATTGCTGTTAG	173
*Fibrobacter succinogenes*	F:GGCGGGATTGAATGTACCTTGAGA R:TCCGCCTGCCCCTGAACTATC	204
*Butyrivibrio fibrisolvens*	F:TAACATGAGAGTTTGATCCTGGCTC R:CGTTACTCACCCGTCCGC	136
*Prevotella ruminicola*	F:CATCCTATAGCGGTAAACCTTTGG R:GAAAGTCGGATTAATGCTCTATGTTG	74
*Ruminobacter amylophilus*	F:CTGGGGAGCTGCCTGAATG R:GCATCTGAATGCGACTGGTTG	100

**Table 3:** Effects of CUR on growth performance in housed growing lambs (n = 12).

Items	Treatments ^a^				SEM	*p*-Value	
	Control	300 CUR	600 CUR	900 CUR		Linear	Quadratic
Initial body weight, kg	20.49	20.96	21.11	20.99	0.23	0.463	0.560
Final body weight, kg	38.12	41.36	40.17	40.12	0.40	0.144	0.030
ADG, g/day	295.24	340.71	315.71	317.86	5.84	0.371	0.050
DMI., g/day	1793.50	1834.76	1858.27	1829.62	20.83	0.501	0.426
DMI/ADG	6.12	5.46	5.97	5.81	0.08	0.497	0.079

^a^ Control, 300 CUR, 600 CUR, and 900 CUR were ram lambs fed the basal diet and supplemented with CUR at a dosage of 0, 300, 600, or 900 mg/kg DM/ day, respectively.

**Table 4:** Effects of CUR on ruminal fermentation in housed growing lambs (n = 12).

Items	Treatments				SEM	*p*-Value	
	Control	300 CUR	600 CUR	900 CUR		Linear	Quadratic
pH	7.13	6.63	6.83	6.89	0.06	0.235	0.008
Total VFA, mmol/L	95.29	110.49	105.44	103.01	2.01	0.280	0.024
Acetate, mmol/L	52.40	60.82	57.34	56.73	0.94	0.199	0.009
Propionate. mmol/L	33.65	38.87	34.39	35.97	0.88	0.504	0.054
Butyrate, mmol/L	10.37	11.91	10.50	9.23	0.45	0.230	0.122
Isovalerate, mmol/L	0.65	0.71	0.67	0.57	0.04	0.445	0.313
Valerate, mmol/L	2.80	3.23	2.54	2.23	0.12	0.023	0.104
Acetate/propionate	1.61	1.42	1.65	1.59	0.06	0.772	0.625
NH_3_-N, mg/dL	12.17	9.42	10.05	11.55	0.37	0.680	0.003

**Table 5:** Effects of CUR on ruminal enzyme activity and microbial population in housed growing lambs (n = 12).

Items	Treatments				SEM	*p*-Value	
	Control	300 CUR	600 CUR	900 CUR		Linear	Quadratic
Microbial enzyme activity							
Cellobiase	0.67	0.75	0.70	0.60	0.02	0.158	0.053
Pectinase	1.55	2.11	1.98	1.72	0.06	0.360	<0.001
Carboxymethyl cellulose	0.31	0.51	0.34	0.31	0.02	0.553	0.013
Xylanase	1.65	2.50	2.49	2.16	0.17	0.314	0.091
Protease	5.10	10.12	8.75	7.58	0.46	0.058	<0.001
α-amylase	0.47	0.79	0.55	0.56	0.06	0.951	0.199
Microflora (copies/mL)							
Total bacteria, ×10^11^	2.85	3.88	3.45	2.69	0.13	0.329	<0.001
Protozoan, ×10^6^	2.13	1.63	1.26	1.17	0.13	0.022	0.383
Methanogens, ×10^8^	1.28	0.97	0.88	0.84	0.06	0.011	0.129
*Ruminococcus albus*, ×10^8^	1.81	3.09	1.82	1.65	0.21	0.555	0.001
*Ruminococcus flavefaciens*, ×10^9^	2.15	2.77	2.65	2.24	0.14	0.892	0.063
*Fibrobacter succinogenes*, ×10^10^	1.64	1.70	1.35	0.91	0.09	<0.001	0.070
*Butyrivibrio fibrisolvens*, ×10^10^	1.29	1.65	1.37	1.14	0.12	0.391	0.118
*Prevotella ruminicola*, ×10^10^	1.41	2.99	2.45	2.67	0.18	0.023	0.032
*Ruminobacter amylophilus*, ×10^8^	1.40	1.78	1.64	1.49	0.08	0.856	0.121

**Table 6:** Effects of curcumin in purine derivatives and microbial protein synthesis in housed growing lambs (n = 6).

Items	Treatments				SEM	*p*-Value	
	Control	300 CUR	600 CUR	900 CUR		Linear	Quadratic
Uric acid, mmol/d	1.00	1.84	1.62	1.44	0.20	0.561	0.228
Allantoin, mmol/d	7.96	12.00	10.96	10.19	0.55	0.182	0.019
Xanthine + hypoxanthine ^a^, mmol/d	0.73	1.12	1.02	0.94	0.06	0.192	0.020
Total PD ^b^, mmol/d	9.68	14.97	13.61	12.57	0.73	0.197	0.021
Exogenous purines absorption ^c^, mmol/d	11.32	17.78	16.12	14.88	0.89	0.197	0.021
MCP ^d^, g/d	51.46	80.79	73.26	67.63	4.06	0.197	0.021

^a^ Xanthine + hypoxanthine = Total PD × 0.075. ^b^ Total PD = (Uric acid + Allantoin)/0.925. ^c^ Exogenous purines absorption, the calculation of X_n_ (Exogenous purines absorption) from Y (Total purine derivatives), X_1_ = Y/0.84, X_(n+1)_ = X_n_ − (0.84X_n_ + 0.150 W^0.75^ e^−0.25Xn^ − Y)/(0.84 − 0.038 W^0.75^ e^−0.25Xn^), where W^0.75^ represents the metabolic body weight (kg) of the lambs. As the iteration process goes on, X_n_ approaches a constant value, which is the exogenous purines absorption. ^d^ MCP = exogenous purines absorption × 70 × 6.25/(0.116 × 0.83 × 1000).

**Table 7:** Effects of curcumin in serum antioxidant capacity in housed growing lambs (n = 12).

Items	Treatments				SEM	*p*-Value	
	Control	300 CUR	600 CUR	900 CUR		Linear	Quadratic
T-AOC, U/mL	18.84	20.88	20.02	18.59	0.27	0.432	0.001
SOD, U/mL	13.05	14.50	13.57	13.11	0.18	0.580	0.004
GSH-Px, U/mL	164.67	182.67	174.96	168.34	2.29	0.857	0.005
CAT, U/mL	73.11	76.96	75.48	74.81	0.60	0.486	0.059
MDA, mmol/mL	5.16	4.80	5.02	4.90	0.04	0.111	0.123

## Data Availability

The datasets generated during the current study are available from the corresponding author on reasonable request.

## References

[1] Low C.X., Tan L.T., Ab M.N., Pusparajah P., Goh B., Chan K., Letchumanan V., Lee L. (2021). Unveiling the impact of antibiotics and alternative methods for animal husbandry: A review. Antibiotics.

[2] Salami S.A., Guinguina A., Agboola J.O., Omede A.A., Agbonlahor E.M., Tayyab U. (2016). Review: In vivo and postmortem effects of feed antioxidants in livestock: A review of the implications on authorization of antioxidant feed additives. Animal.

[3] Zhong R.Z., Zhou D.W. (2013). Oxidative stress and role of natural plant derived antioxidants in animal reproduction. J. Integr. Agric..

[4] Rege S.A., Arya M., Momin S.A. (2019). Structure activity relationship of tautomers of curcumin: A review. Ukr. Food J..

[5] Fadhlurrahma A., Saepudin E., Rahayu D.U.C. (2020). Acetylation of curcuminoids extract from Turmeric Rhizomes (*Curcuma longa*) as antibacterial compounds against *S. aureus* and *E. coli*. IOP Conf. Ser. Mater. Sci. Eng..

[6] Guo J., Zhang Y.Y., Sun M., Xu L.F. (2022). Therapeutic potential of curcumin in a rat model of dextran sulfate sodium-induced ulcerative colitis by regulating the balance of tregth17 cells. Inflammation.

[7] Pontes Q.G.M., Benito G.L., Cano J.P., Aguilar M.R., Vázquez L.B. (2020). Amphiphilic polymeric nanoparticles encapsulating curcumin: Antioxidant, anti-inflammatory and biocompatibility studies. Mater. Sci. Eng. C.

[8] Orzuna-Orzuna J.F., Dorantes-Iturbide G., Lara-Bueno A. (2021). Effects of dietary tannins’ supplementation on growth performance, rumen fermentation, and enteric methane emissions in beef cattle: A metaanalysis. Sustainability.

[9] Jafari S., Ebrahimi M., Goh Y.M. (2019). Manipulation of rumen fermentation and methane gas production by plant secondary metabolites (saponin, tannin and essential oil): A review of ten-year studies. Ann. Anim. Sci..

[10] Silva S.N.S.E., Chabrillat T., Kerros S. (2021). Effects of plant extract supplementations or monensin on nutrient intake, digestibility, ruminal fermentation and metabolism in dairy cows. Anim. Feed Sci. Technol..

[11] Vasta V., Daghio M., Cappucci A. (2019). Invited review: Plant polyphenols and rumen microbiota responsible for fatty acid biohydrogenation, fiber digestion, and methane emission: Experimental evidence and methodological approaches. J. Dairy Sci..

[12] Marcon H., Souza C.F., Baldissera M.D., Alba D.F., Favaretto J.A., Santos D.S., Borges L., Kessler J.D., Vedovatto M., Bianchi A.E. (2021). Effect of curcumin dietary supplementation on growth performance, physiology, carcass characteristics and meat quality in lambs. Ann. Anim. Sci..

[13] Jiang Z.Y., Wan Y.G., Li P., Xue Y., Cui W.W., Chen Q., Chen J.Q., Wang F., Mao D.G. (2019). Effect of curcumin supplement in summer diet on blood metabolites, antioxidant status, immune response, and testicular gene expression in hu sheep. Animals.

[14] Wang C., Liu Q., Guo G., Huo W.J., Ma L., Zhang Y.L., Pei C.X., Zhang S.L., Wang H. (2016). Effects of rumen-protected folic acid on ruminal fermentation, microbial enzyme activity, cellulolytic bacteria and urinary excretion of purine derivatives in growing beef steers. Anim. Feed Sci. Technol..

[15] Agarwal N., Kamra D.N., Chaudhary L.C., Agarwal L., Sahoo A., Pathak N.N. (2002). Microbial status and rumen enzyme profile of crossbred calves fed on different microbial feed additives. Lett. Appl. Microbiol..

[16] Chen X.B., Gomes M.J. (1992). Estimation of Microbial Protein Supply to Sheep and Cattle Based on Urinary Excretion of Purine Derivatives—An Overview of Technical Details.

[17] Yu Z.T., Morrison M. (2004). Improved extraction of PCR-quality community DNA from digesta and fecal samples. Biotechniques.

[18] Kongmun P., Wanapat M., Pakdee P., Navanukraw C. (2010). Effect of coconut oil and garlic powder on in vitro fermentation using gas production technique. Livest. Sci..

[19] Wu H.M., Zhang J., Wang C., Liu Q., Guo G., Huo W.J., Chen L., Zhang Y.L., Pei C.X., Zhang S.L. (2021). Effects of riboflavin supplementation on performance, nutrient digestion, rumen microbiota composition and activities of Holstein bulls. Br. J. Nutr..

[20] Feng T., Hu Z.S., Wang K., Wang K., Zhu X., Chen D., Zhuang H.N., Yao L.Y., Song S.Q., Wang H.T. (2020). Emulsion-based delivery systems for curcumin: Encapsulation and interaction mechanism between debrancher starch and curcumin. Int. J. Biol. Macromol..

[21] Alam J., Dilnawaz F., Sahoo S.K., Singh D.V., Mukhopadhyay A.K., Hussain T., Pati S. (2022). Curcumin encapsulated into biocompatible Co-polymer PLGA nanoparticle enhanced anti-gastric cancer and anti-helicobacter pylori effect. Asian Pac. J. Cancer Prev..

[22] Florian T., Johann S., Lothar B. (2020). Spectroscopic studies on the molecular interactions of curcumin and piperine. Monatsh Chem..

[23] Wang Y., Lu Z.X., Wu H., Lv F.X. (2009). Study on the antibiotic activity of microcapsule curcumin against foodborne pathogens. Int. J. Food Microbiol..

[24] Luo D.D., Chen J.F., Liu J.J., Xie J.H., Zhang Z.B., Gu J.Y., Zhuo J.Y., Huang S., Su Z.R., Sun Z.H. (2018). Tetrahydrocurcumin and octahydrocurcumin, the primary and final hydrogenated metabolites of curcumin, possess superior hepatic-protective effect against acetaminophen-induced liver injury: Role of CYP2E1 and Keap1-Nrf2 pathway. Food Chem. Toxicol..

[25] Lopresti A.L. (2018). The Problem of Curcumin and Its Bioavailability: Could Its Gastrointestinal Influence Contribute to Its Overall Health-Enhancing Effects?. Adv. Nutr..

[26] Vorlaphim T., Phonvisay M., Khotsakdee J., Vasupen K., Bureenok S., Wongsuthavas S., Alhaidary A., Mohamed H.E., Beynen A.C., Yuangklang C. (2011). Influence of dietary curcumin on rumen fermentation, macronutrient digestion and nitrogen balance in beef cattle. Am. J. Agric. Biol. Sci..

[27] Antonise M., Jaguezeski G.P., Nathieli B., Bottari R.W., Mariane B., Fagundes M.R.C., Schetinger V.M., Morsch C.S., Stein R.N., Moresco D.A. (2018). Addition of curcumin to the diet of dairy sheep improves health, performance and milk quality. Anim. Feed Sci. Technol..

[28] Nozière P., Glasser F., Sauvant D. (2010). In vivo production and molar percentages of volatile fatty acids in the rumen: A quantitative review by an empirical approach. Animal.

[29] Xun W.J., Shi L.G., Zhou H.L., Hou G.Y., Cao T., Zhao C.P. (2015). Effects of curcumin on growth performance, jejunal mucosal membrane integrity, morphology and immune status in weaned piglets challenged with enterotoxigenic Escherichia coli. Int. Immunopharmacol..

[30] Zhong R.Z., Yu M., Liu H.W., Sun H.X., Cao Y., Zhou D.W. (2012). Effects of dietary Astragalus polysaccharide and Astragalus membranaceus root supplementation on growth performance, rumen fermentation, immune responses, and antioxidant status of lambs. Anim. Feed Sci. Technol..

[31] Zhou L.L., Wang D.F., Hu H.C., Zhou H.L. (2020). Effects of Piper sarmentosum extract supplementation on growth performances and rumen fermentation and microflora characteristics in goats. J. Anim. Physiol. Anim. Nutr..

[32] Akito S., Nose K., Takaoka M., Hayashi H., Kond T. (2009). Effect of dietary turmeric on breath hydrogen. Dig. Dis. Sci..

[33] Li S.Y., Wang C., Wu Z.Z., Liu Q., Guo G., Huo W.J., Zhang J., Chen L., Zhang Y.L., Pei C.X. (2020). Effects of guanidinoacetic acid supplementation on growth performance, nutrient digestion, rumen fermentation and blood metabolites in Angus bulls. Anim. Int. J. Anim. Biosci..

[34] Vitor M., Carine F.S., Matheus D. (2018). Diet supplemented with curcumin for nursing lambs improves animal growth, energetic metabolism, and performance of the antioxidant and immune systems. Small Rumin. Res..

[35] Vogels G.D., Hoppe W.F., Stumm C.K. (1982). Association of methanogenic bacteria with rumen ciliates. Appl. Environ. Microbiol..

[36] Dohme F., Machmüller A., Estermann B.L., Pfister P., Wasserfallen A., Kreuzer M. (1999). The role of the rumen ciliate protozoa for methane suppression caused by coconut oil. Lett. Appl. Microbiol..

[37] Feng W., Wang H., Zhang P., Gao C., Tao J., Ge Z., Zhu D., Bi Y. (2017). Modulation of gut microbiota contributes to curcumin-mediated attenuation of hepatic steatosis in rats. Biochim. Biophys. Acta.

[38] Bereswill S., Munoz M., Fischer A., Plickert R., Haag L.M., Otto B., Kuhl A.A., Loddenkemper C., Gobel U.B., Heimesaat M.M. (2010). Antiinflammatory effects of resveratrol, curcumin and simvastatin in acute small intestinal inflammation. Public Libr. Sci. One.

[39] Mu C.T., Yang W.J., Wang P.J., Zhao J.X., Hao X.Y., Zhang J.X. (2020). Effects of high-concentrate diet supplemented with grape seed proanthocyanidins on growth performance, liver function, meat quality, and antioxidant activity in finishing lambs. Anim. Feed Sci. Technol..

[40] Weimer P.J., Hatfield R.D., Buxton D.R. (1993). Inhibition of ruminal cellulose fermentation by extracts of the perennial legume cicer milkvetch (Astragalus cicer). Appl. Environ. Microbiol..

[41] Boucher S.E., Ordway R.S., Whitehouse N.L., Lundy F.P., Kononoff P.J., Schwab C.G. (2007). Effect of incremental urea supplementation of a conventional corn silage-based diet on ruminal ammonia concentration and synthesis of microbial protein. J. Dairy Sci..

[42] Firkins J.L., Yu Z., Morrison M. (2007). Ruminal nitrogen metabolism: Perspectives for integration of microbiology and nutrition for dairy. J. Dairy Sci..

[43] Descalzo A.M., Sancho A.M. (2008). A review of natural antioxidants and their effects on oxidative status, odor and quality of fresh beef produced in Argentina. Meat Sci..

[44] Cao S.T., Wang C.C., Yan J.T., Li X., Wen J.S., Hu C.H. (2020). Curcumin ameliorates oxidative stress-induced intestinal barrier injury and mitochondrial damage by promoting Parkin dependent mitophagy through AMPK-TFEB signal pathway. Free Radic. Biol. Med..

[45] Zhang L.G., Zhang J.Q., Yan E.F., He J.T., Zhong X., Zhang L.L., Wang C., Wang T. (2020). Dietary Supplemented Curcumin Improves Meat Quality and Antioxidant Status of Intrauterine Growth Retardation Growing Pigs via Nrf2 Signal Pathway. Animals.

[46] Galati G., Sabzevari O., Wilson J.X., O’Brien P.J. (2002). Prooxidant activity and cellular effects of the phenoxyl radicals of dietary flavonoids and other polyphenolics. Toxicology.

